# Safety and effectiveness of tigecycline combination therapy in renal transplant patients with infection due to carbapenem-resistant gram-negative bacteria

**DOI:** 10.3389/fcimb.2023.1215288

**Published:** 2023-11-14

**Authors:** Qin Wang, Guiyi Liao, Quan Xia, Chaoliang Ge, Handong Ding

**Affiliations:** ^1^ Departent of Urology, the First Affiliated Hospital of Anhui Medical University, Hefei, Anhui, China; ^2^ Institute of Urology, Anhui Medical University, Hefei, Anhui, China; ^3^ Anhui Province Key Laboratory of Genitourinary Diseases, Anhui Medical University, Hefei, Anhui, China; ^4^ Department of Pharmacy, the First Affiliated Hospital of Anhui Medical University, Hefei, Anhui, China; ^5^ The Grade 3 Pharmaceutical Chemistry Laboratory of State Administration of Traditional Chinese Medicine, Hefei, Anhui, China

**Keywords:** tigecycline, renal transplant recipient, carbapenem-resistant gram-negative bacteria, acute pancreatitis, interaction

## Abstract

**Background:**

Carbapenem-resistant gram-negative bacterial (CRGNB) infections are increasing among kidney transplant recipients, and effective therapeutic options are limited. This study aimed to investigate the efficacy and adverse events associated with combination therapy tigecycline in renal transplant patients with CRGNB infections.

**Methods:**

This study retrospectively analyzed 40 Chinese patients with confirmed or suspected CRGNB infections who received tigecycline therapy. The patients’ case features and clinical and microbiological data were analyzed.

**Results:**

A total of 40 renal transplant recipients received tigecycline therapy for a median duration of 9 (range, 3–25) days. CRGNB isolates were obtained from the organ preservation solution of the donor kidney in 28 patients, with confirmed transmission in 4 patients. Infections were detected in the bloodstream, urinary tract, sputum, and wound. The most prevalent isolates were *Klebsiella pneumoniae* (75%, 30/40), *Acinetobacter baumannii* (15%, 6/40), and *Escherichia coli* (10%, 4/40). A clinical response was observed in 32 (80%) patients. The 28-day all-cause mortality rate was 7.5% (3/40), while the one-year all-cause mortality rate was 2.5% (1/40). While one patient died owing to severe pancreatitis, no serious adverse events related to tigecycline therapy were reported. However, multiple indices of liver function and pancreatitis precursors increased after treatment with tigecycline compared to before treatment.

**Conclusion:**

Tigecycline therapy appears to be well tolerated in renal transplant recipients with multidrug-resistant bacterial infections. Nevertheless, attention should be paid to adverse reactions related to tigecycline therapy, especially gastrointestinal reactions, and the related laboratory tests should be closely monitored.

## Introduction

Carbapenem-resistant gram-negative bacterial (CRGNB) infections often occur in kidney transplant patients owing to the use of immunosuppressants and broad-spectrum antibiotics. A large proportion of these infections arise from donation after cardiac death (DCD). Most of these patients develop CRGNB infections due to long-term hospitalization and the use of broad-spectrum antibiotics, increasing the risk of pathogen transmission during transplantation. The treatment of CRGNB infections in these patients poses challenges, primarily due to the limited availability of effective antibiotics and a lack of systematic evaluation of antibiotic pharmacokinetics in the transplant population. CRGNB infections are associated with elevated mortality rates, increased morbidity, extended hospital stays, and higher healthcare costs, especially when combined with intense immunosuppressive therapy in the early stages of transplantation (Bergamasco et al., 2012; Mularoni et al., 2015). Antibiotic resistance, especially among gram-negative pathogens, has been increasing, leaving them susceptible to only a few antibiotics, such as tigecycline (TGC). TGC is a tetracycline-based antibacterial agent that exhibits efficacy against a variety of multidrug-resistant gram-negative bacteria and gram-positive bacteria (Hurtado et al., 2012; Ozdemir et al., 2012; Lin et al., 2016). The use of TGC in combination with other antimicrobial agents for the treatment of resistant *Acinetobacter baumannii* and *Enterobacter* has been validated and proposed in guidelines for treating infections. However, owing to the unique pathophysiological state and low immunity of renal transplant patients, the safety and effectiveness of TGC in these patients remain unclear. At present, there are few studies on the efficacy and adverse events of TGC in renal transplant patients, mostly comprising case reports and small sample case series.

Therefore, this study aimed to evaluate the effectiveness and safety of TGC in renal transplant recipients and report a case of acute pancreatitis possibly caused by TGC.

## Patients and methods

### Study design

This retrospective study comprising renal transplant recipients was conducted at the First Affiliated Hospital of Anhui Medical University between June 2019 and December 2021. A total of 40 patients were eligible for inclusion. Once CRGNB infection was detected from the culture of an organ preservation solution and/or specimens derived from patients, the patient immediately received TGC in combination with other antibiotics (carbapenems or cefoperazone–sulbactam) for at least three days after renal transplantation. Pathogen cultures from the urinary tract, wound, bloodstream, and respiratory specimens were routinely collected from donors and recipients. Prior to transplantation, organ preservation fluid samples were sent for microbial culture. The study procedures were approved by the Ethics Committee at the First Affiliated Hospital of Anhui Medical University.

### Data collection

We created a database to collect patient information, including age, sex, laboratory test tests (amylase and lipase, etc.), details of TGC treatment (administration route, dosage, and treatment course), concomitant use of other antibiotics if applicable, infection sites (bloodstream and respiratory tract), outcomes of TGC treatment (including presenting symptoms, recurrence of infection, clinical efficacy, and mortality), and documentation of adverse events.

### Microbiology

Susceptibility to TGC was tested using the disk diffusion method (**Supplementary Tables S1**-**S3**). A bacterial isolate was considered susceptible to TGC if the inhibition zone was ≥19 mm, intermediate if it was 13–18 mm, and resistant if was ≤14 mm, according to the FDA breakpoint criteria (Hui et al., 2020). The susceptibility of bacterial isolates to other antimicrobial agents was determined using the VITEK-2 system (Biomerieux, Marcy-l ‘Etoile, France). CRGNB infections were defined as isolates resistant to meropenem or imipenem, with a minimum inhibitory concentration (MIC) ≥4 μg/mL, according to the 2023 Clinical and Laboratory Standards Institute guidelines.

### Anti-infection regimen

When CRGNB infection was detected in the organ preservation solution, recipients, and/or donors, the patients were immediately administered TGC (50 mg, q12h, intravenous injection (ivgtt)) in combination with other antibiotics. All isolated CRGNB strains were sensitive to TGC. The patients included in this study were aged ≥18 years and had been administered intravenous TGC for ≥3 days. The dosage regimen for TGC involved a loading dose of 100 mg followed by a maintenance dose of 50 mg every 12 hours. The dosing of other antimicrobial agents was adjusted based on renal function, as recommended by the manufacturer.

### Adverse events

During the use of TGC, patients were closely monitored for adverse events, including symptoms such as allergic reactions, gastrointestinal reactions, and acute pancreatitis. In addition, laboratory tests were conducted to assess indicators such as jaundice, abnormal liver function, hyperbilirubinemia, and cholestasis (Gilson et al., 2008; Tanaseanu et al., 2008; Lin et al., 2018).

### Statistical analysis

Statistical analysis was conducted using the SPSS 26.0 statistical package (IBM, Armonk, NY, USA). Continuous variables were presented as mean ± standard deviation or median and interquartile range and compared using Student’s *t*-test or the Mann–Whitney *U*-test, as appropriate. Paired data were analyzed using paired Student’s *t-*test or the Wilcoxon matched test. *P* < 0.05 was considered to indicate a statistically significant difference.

## Results

### Clinical and microbiological characteristics

In total, 40 patients (10 female) with a median age of 39 (range, 32–49) years received TGC treatment for CRGNB infections. *Klebsiella pneumoniae* accounted for 75% (30/40), *Acinetobacter baumannii* accounted for 15% (6/40), and *Escherichia coli* accounted for 10% (4/40) of the strains isolated from the donors or recipients. All isolated strains had imipenem minimum inhibitory concentrations (MICs) of ≥8 mg/L. A total of 28 strains of CRGNB were isolated from organ preservation cultures, and among these, 5 strains may have been transmitted infections. In addition, other infections originated from the recipients and included bloodstream infections in 4 patients, urinary tract infections in 2 patients, respiratory tract infections in 3 patients, and wound infections in 2 patients. All patients were treated with TGC for at least three days. Among the 40 patients, 12 developed septic shock. Detailed information on the patients is shown in **Table 1**.

**Table 1:** Demographic and clinical data of renal transplant recipients treated with tigecycline for carbapenem-resistant gram-negative bacteria.

Characteristics		Value
Sex (Male/Female)		30/10
Age (year)-median (IQR)		39 (32-49)
Septic shock		12 (30%)
Bacterial source	Organ preservation solutions	28 (70%)
	Urinary tract	2 (5%)
	Sputum	3 (7.5%)
	Wound	2 (5%)
	Bloodstream	5 (12.5%)
Pathogen causing bacteremia	*Klebsiella pneumoniae*	30 (75%)
	*Acinetobacter baumannii*	6 (15%)
	*Escherichia coli*	4 (10%)

### Treatment regimens and outcomes

*In vitro* testing showed that all isolated strains were sensitive to TGC but resistant to meropenem or imipenem. All patients received combination therapy, with 34 receiving meropenem or imipenem cilastatin + TGC and 6 receiving cefoperazone–sulbactam + TGC. There was no difference among patients treated with carbapenem or cefoperazone–sulbactam in combination with tigecycline. The median duration of treatment with TGC was 9 (range, 6–14) days. The clinical cure rate with TGC treatment was 80% (32/40); 20% (8/40) of patients failed to respond to TGC therapy and required a second-line treatment with ceftazidime–avibactam. The clinical features of these 8 patients are listed in **Table 2**. The 28-day all-cause mortality rate was 7.5% (3 patients), with 1 patient developing an uncontrollable *A. baumannii* infection, another potentially developing pancreatitis, and another potentially dying from a myocardial infarction. Moreover, 1 (2.5%) patient died within one year due to cytomegalovirus infection. The results are shown in **Table 3**. A total of 37 patients were tested for procalcitonin (PCT). After treatment with TGC, PCT levels significantly reduced compared to before treatment (Z = −3.523, *P* < 0.001; **Table 4**).

**Table 2:** Patients treated with ceftazidime–avibactam.

NO.	Infection site	Pathogen	Duration	Clinical outcome
1	Organ preservation solutions	*Klebsiella pneumoniae*	7	Improved
2	Organ preservation solutions	*Escherichia coli*	10	Died
3	Organ preservation solutions	*Klebsiella pneumoniae*	11	Improved
4	Organ preservation solutions	*Acinetobacter baumannii*	10	Improved
5	Organ preservation solutions+Bloodstream	*Klebsiella pneumoniae*	10	Improved
6	Organ preservation solutions+Bloodstream	*Klebsiella pneumoniae*	11	Improved
7	Organ preservation solutions	*Klebsiella pneumoniae*	Died	Died
8	Bloodstream	*Klebsiella pneumoniae*	9	Improved

**Table 3:** Anti-infective treatments and outcomes.

Characteristics		Value
Combination therapy in addition to tigecycline	Meropenem Cefoperazone–sulbactam	34 (85%) 6 (15%)
Duration of tigecycline (day)-median (IQR)		9 (6-14)
Therapeutic regimen Loading dose Maintenance dose	100 mg 50 mg	40 (100%) 40 (100%)
Antibiotic regimens after tigecycline therapy	Ceftazidime–avibactam 2.5g qd-2.5q q8h	8 (20%)
Outcome	Death within 28 days	3 (7.5%)
	Death within 1year	1 (2.5%)
Presenting symptoms	Nausea, vomiting, abdominal distension, abdominal pain	14 (35%)
	None	12 (65%)

**Table 4:** Laboratory tests of patients before and after tigecycline treatment.

Variable	Before tigecycline treatment	After tigecycline treatment	*P*
PCT (ng/ml) (n=37)	5 (2.05, 18.85)	1.57 (0.4, 4.135)	Z=-3.523 *P*<0.001
Amylase (U/L) (n=30)	93 (41.25, 146.5)	114.5 (69.5, 260.8)	Z=-3.276 *P*=0.001
Lipase (U/L) (n=30)	163 (75.2, 304.5)	425 (153.25, 1132.8)	Z=-4.076 *P*<0.001
ALT (U/L) (n=35)	22 (15, 29)	24 (18, 39)	Z=-2.091 *P*=0.037
AST (U/L) (n=35)	25 (17, 39)	26 (17, 58)	Z=-2.666 *P*=0.008
ALP (U/L) (n=35)	104 (51, 153)	137 (106, 234)	Z=-2.163 *P=0.031*
γ-GGT (U/L) (n=35)	29 (13, 110)	56 (24, 128)	Z=-1.028 *P=0.304*
TB (umol/L) (n=35)	10 (7.6, 14)	17.7 (10.6, 36)	Z=-3.761 *P*<0.001

ALP, alkaline phosphatase; AST, aspartate aminotransferase; ALT, alanine aminotransferase; γ-GGT, gamma-glutamine amide transpeptidase; PCT, procalcitonin; TB, total bilirubin.

### Adverse events

Except for 1 patient who died from severe pancreatitis, no other serious adverse reactions were observed in the patients. However, clinical gastrointestinal symptoms were reported by a considerable proportion of patients, with 28 out of 40 (35%) patients experiencing nausea, vomiting, abdominal distension, and/or abdominal pain (**Table 3**). Before and after the use of TGC, multiple indices of liver function and pancreatitis precursors were examined (**Table 4**). A total of 30 patients were tested for amylase and lipase levels, and the results showed a significant increase in lipase and amylase levels after treatment (Z = −3.276, *P* = 0.001 and Z = −4.076, *P* < 0.001) (**Table 4**). Biochemical indicators reflecting liver toxicity, including aspartate aminotransferase (AST), alanine aminotransferase (ALT), gamma-glutamine amide transpeptidase (γ-GGT), alkaline phosphatase (ALP), and total bilirubin (TB) were assessed in 35 patients. The results showed an increase in ALT, AST, ALP, γ-GGT, and TB levels after treatment with TGC compared to before treatment with TGC (**Table 4**). There were no differences in biochemical parameters between the combination treatments.

### Special case

The patient was a 44-year-old female diagnosed with chronic glomerulonephritis who had been undergoing regular dialysis since February 2015. She had a history of hypertension for more than 20 years, which had been well controlled with nifedipine sustained-release tablets. In January 2020, the patient underwent a successful renal transplant from a DCD donor. Postoperative medications included a triple immunosuppressive regimen of tacrolimus (FK-506), mycophenolate mofetil, and glucocorticoids. After surgery, the patient’s urine output and creatinine levels recovered well. Her serum tacrolimus level was 5.3 ng/mL three days after the transplant. However, on the eighth day, the serum tacrolimus level elevated to over 30 ng/mL. On the third day after transplantation, carbapenem-resistant *K. pneumoniae* was detected in the organ preservation solution. No positive bacteria were found in urine, drainage fluid, or blood cultures. Subsequently, the patient received 50 mg of TGC every 12 h (100 mg for the first dose) + meropenem 0.5 g every 8 h for eight days. On the fourth day after transplantation, the patient experienced nausea, vomiting, abdominal distension, and abdominal pain. Emergency abdominal CT showed no obvious abnormality. These symptoms lasted for several days without significant exacerbation. Emergency B-ultrasound of the upper abdomen on the ninth day did not detect any abnormality, but laboratory results showed elevated serum levels of lipase (665 [reference range, 23−300] U/L) and amylase (157 [reference range, 30−100] U/L). Subsequently, the patient was diagnosed with acute pancreatitis (AP) and provided conservative treatment. On the tenth day, the patient’s abdominal pain and distension worsened, and active abdominal hemorrhage occurred. Her blood pressure continued to decline despite receiving blood transfusion and fluids. Consequently, an emergency exploratory laparotomy was performed, which revealed hemorrhaging and necrosis in the pancreatic tissue, confirming the diagnosis of AP. Most of the necrotic tissue was removed during the surgery. The postoperative abdominal drainage tube drained hemorrhagic fluid, and the patient died of hemorrhagic shock the next day.

## Discussion

Approximately 10% to 20% of patients who undergo solid organ transplantation are infected with multidrug-resistant gram-negative bacteria, with carbapenem-resistant *K. pneumoniae* (CRKP) being the predominant pathogen (Bodro et al., 2013). The rate of CRKP infection after kidney transplantation is around 5% (Bodro et al., 2013; Simkins et al., 2014; Pouch et al., 2015). Studies have shown that kidney transplant recipients infected with CRKP experience graft loss in 15%–20% of cases (Lanini et al., 2015; Pouch et al., 2015). In our study, the infections were mainly derived from the donors. Some studies have reported several cases of donor-derived infection with poor prognoses. For instance, Simkins et al. reported 13 patients with CRKP infection, of whom 2 (15%) experienced graft loss and 6 (46%) died (Simkins et al., 2014). Another study reported 5 patients with donor-derived CRKP infection, 3 of whom died (Bergamasco et al., 2012). In China, Wang et al. reported eight cases of donor-derived CRKP infection after renal transplantation, which were treated with TGC and extended-infusion meropenem. Among them, 7 patients were successfully cured, whereas 1 patient died of septic shock (Wang et al., 2021). Another study from China showed a high mortality rate (4/5) in cases of donor-derived CRKP bloodstream infection (Wang et al., 2018). In our study, 32 (80%) patients were cured with the combination therapy containing TGC, whereas 3 (7.5%) patients died within 28 days. The different cure rates might be attributed to the different treatment regimens and the difference in the severity of the infections. Based on the results of the drug sensitivity analysis, *K. pneumoniae* was sensitive only to ceftazidime–avibactam, TGC, and minocycline. However, owing to the higher cost of ceftazidime–avibactam therapy, the patients were more inclined to opt for the relatively cheaper TGC and minocycline. TGC is generally not used alone and is often combined with antimicrobial agents. The results of *in vitro* drug sensitivity analysis indicated that *A. baumannii* was sensitive to TGC and minocycline while showing intermediate sensitivity to cefoperazone–sulbactam. Therefore, a combination of TGC and cefoperazone–sulbactam was used to treat infections caused by *A. baumannii*. Minocycline plus cefoperazone–sulbactam can also be used for these infections.

TGC has become one of the therapeutic options for CRGNB infections. However, there have been concerns regarding its safety and efficacy. In 2010 and 2013, according to a meta-analysis based on randomized clinical trials, the US Food and Drug Administration (FDA) warned that the use of TGC in adults was associated with increased mortality (Bassetti et al., 2014). Moreover, a retrospective study reported that the overall mortality rate was 60.7% in 84 patients treated with TGC for pneumonia caused by multidrug-resistant A. *baumannii*. The high mortality observed in the group of patients treated with TGC may be associated with higher MICs of TGC (Chuang et al., 2014). Nevertheless, in real-world clinical care, off-label TGC is particularly useful for infections caused by MDR/XDR bacteria in critically ill adult patients (De Pascale et al., 2014; Falagas et al., 2014b; De Pascale et al., 2020). Another meta-analysis and systematic review reported that increasing the dose of TGC was safe and effective (Gong et al., 2019; Zha et al., 2020). Furthermore, a recent Japanese study showed that TGC was associated with a 59% cure rate in the treatment of CRGNB (Ohashi et al., 2022). However, the safety and efficacy of TGC in special populations, such as kidney transplant patients, is not yet fully understood. There are differences in the safety and efficacy of TGC in different patients, which may arise from variations in the pharmacokinetic–pharmacodynamic profiles, site of infection, and dosing.

In patients with renal impairment, conventional doses of TGC (50 mg every 12 h) were effective for intra-abdominal infections and hospital-acquired pneumonia (HAP) caused by *E. coli* and gram-positive bacteria. For HAP caused by *K*. *pneumoniae* and *Enterobacter cloacae*, patients with severe renal failure can use the conventional dose, whereas other patients may need an increased dose of TGC (Li et al., 2020). Fan et al. reported that the safety and efficacy of TGC in ICU patients should be based on therapeutic drug monitoring, and a cut-off value of 474.8 ng/mL of trough plasma concentrations (C_min_) can be used as a predictor of hepatotoxicity (Fan et al., 2020). The pharmacokinetics/pharmacodynamics of TGC in renal transplant patients remain unclear, but they may have a role in determining its safety and efficacy in these patients. In the Chinese population, most patients tolerated the combined therapy (TGC and extended-infusion meropenem) well (Wang et al., 2021).

The use of TGC has been associated with various adverse effects, including gastrointestinal reactions, acute pancreatitis, allergic reactions, abnormal liver function, jaundice, cholestasis, hyperbilirubinemia, and superinfection. Compared to other treatment regimens, TGC treatment regimens have been found to be associated with a higher incidence of gastrointestinal adverse reactions, with nausea and vomiting being the most commonly reported adverse effects (Zha et al., 2020). In recent years, increased attention has been paid to TGC-induced AP. After post-marketing surveillance in 2006, AP was considered an adverse reaction associated with the use of TGC (Okon et al., 2013). The characteristics of AP are abdominal distension, abdominal pain, elevated amylase and lipase levels, and edematous infiltrate.

There have been some case reports of TGC-induced AP (Fang et al., 2020). After renal transplantation, only three cases of AP caused by TGC or FK-506 have been reported: (1) A patient who was treated with TGC for perianal cellulitis developed AP on the tenth day of treatment. After discontinuation of TGC, the patient experienced a second episode of AP on the third day of re-treatment for a complicated abdominal infection (Yazirli et al., 2021). (2) A woman developed a donor-derived carbapenem-resistant *A. baumannii* (CRAB) infection. After 15 days of treatment with TGC, the woman developed AP with symptoms, relevant CT findings, and elevated pancreatic enzymes (Lin et al., 2018). (3) A 61-year-old man infected with non-tuberculous *Mycobacterium chelonae* developed AP after four weeks of TGC therapy. His symptoms resolved within 24–48 hours after discontinuation of the medication (Akhter et al., 2018). Another case report showed that a kidney transplant patient developed AP after receiving both FK-506 and TGC, with the author suggesting that FK-506 was the cause of AP (Liu et al., 2021).

In the present study, one patient died of AP. Unlike the previous case reports, the pathogens in our study were mainly CRKP and CRAB, most of which came from the organ preservation solution. The lipase and amylase levels of 30 patients were significantly elevated after treatment with TGC compared to before treatment with TGC. Liver function tests showed increased levels of ALT, AST, ALP, γ-GGT, and TB after treatment with TGC compared to before treatment with TGC, with a more pronounced elevation observed in ALP, γ-GGT, and TB. Hepatotoxicity caused by TGC has been reported, although the underlying mechanisms are still unclear (Ellis-Grosse et al., 2005; Kadoyama et al., 2012; Cheng Gy et al., 2018). In one study, the incidence of gastrointestinal adverse effects was positively correlated with the dose of TGC (Falagas et al., 2014a). However, other studies have shown no adverse effects in patients receiving high-dose TGC therapy compared to standard doses (De Pascale et al., 2014; Kim et al., 2014). To the best of our knowledge, this is the first study on adverse effects associated with TGC treatment in renal transplant patients, other than some case reports. The observed differences may be due to different physiological states, such as kidney transplant patients, renal insufficiency patients, severe patients, and infection sites. Therefore, individualized dosing and assessment may be required for different disease states.

It has been reported that FK-506 may interact with TGC, and the use of TGC may lead to an elevated serum FK-506 concentration, which subsequently decreases after discontinuation of TGC (Pavan et al., 2011; Chow et al., 2020; Yazirli et al., 2021). FK-506 is metabolized by the cytochrome P450 3A4 (CYP3A4) enzyme, and TGC is not a substrate, inhibitor, or inducer of the common cytochrome P450 enzyme. Therefore, TGC may interact with FK-506 through other mechanisms, such as their shared substrate role for the membrane transporter p-glycoprotein (p-gp). In our study, the serum FK-506 level reached the maximum concentration (>30 [range, 5–20] ng/mL) on the sixth day of administration of TGC. Meanwhile, the FK-506 dosage was adjusted from 2.5 mg twice daily to 1 mg twice daily. On the ninth day, after discontinuation of TGC, the serum FK-506 concentration was no longer detected. Therefore, it was not possible to definitively determine whether FK-506 interacted with TGC in this case.

## Conclusion

Our findings suggest that combination therapy with TGC may be an option for treating CRGNB infections after renal transplantation. Although TGC could be tolerated well by Chinese kidney transplant patients, attention should be paid to potential adverse reactions such as gastrointestinal complications, hepatotoxicity, and TGC-induced AP. Clinicians should pay attention to the clinical symptoms and the serum amylase and lipase levels of kidney transplant patients. Further research is needed to determine the potential interaction between TGC and FK-506.

## Data availability statement

The original contributions presented in the study are included in the article/**Supplementary Material**. Further inquiries can be directed to the corresponding author.

## Ethics statement

The studies involving humans were approved by the Ethics Committee at the First Affiliated Hospital of Anhui Medical University. The studies were conducted in accordance with the local legislation and institutional requirements. Given the non-interventional nature, informed consent was not required. Written informed consent was obtained from the individual(s) for the publication of any potentially identifiable images or data included in this article.

## Author contributions

QW and HD are responsible for the conception and design, development of methodology, data analysis and interpretation, writing, review, and revision of the manuscript. GL, QX, and CG contributed to the study concept and design and interpretation of the data. All authors contributed to the article and approved the submitted version.
