# Supplementary material for: Safety and effectiveness of tigecycline combination therapy in renal transplant patients with infection due to carbapenem-resistant gram-negative bacteria
Source: Front Cell Infect Microbiol. 2023 Nov 14;13:1215288. doi: 10.3389/fcimb.2023.1215288 (PMC10682949; doi:10.3389/fcimb.2023.1215288)
Supplement: Supplementary file 1 [file Table_1.doc]

**Table S1.** Antimicrobial susceptibility of *Acinetobacter baumannii*

| **Antibiotics** | **K-B (mm)** | **MIC/μg.ml^-1^** | **Results** |
| --- | --- | --- | --- |
| Ceftazidime |  | >=64.0 | R |
| Ceftriaxone |  | >=64.0 | R |
| Gentamicin |  | >=16.0 | R |
| Ciprofloxacin |  | >=4.0 | R |
| Compound sulfamethoxazole |  | >=320.0 | R |
| Tobramycin |  | >=16.0 | R |
| Cefepime |  | >=64.0 | R |
| Imipenem |  | >=16.0 | R |
| Levofloxacin |  | >=8.0 | R |
| Ampicillin-sulbactam |  | >=16.0 | R |
| Cefazolin |  | >=64.0 | R |
| Piperacillin | 6 |  | R |
| Cefotaxime | 6 |  | R |
| Meropenem | 9 |  | R |
| Cefoperazone-sulbactam | 18 |  | I |
| Minocycline | 21 |  | S |
| Tigecycline | 21 |  | S |
| Amikacin | 6 |  | R |
| Piperacillin-tazobactam | 6 |  | R |

S: susceptible; R: resistant； I: intermediate；MIC: minimum inhibitory concentration.

**Table S2.** Antimicrobial susceptibility of *Klebsiella pneumoniae*

| **Antibiotics** | **K-B (mm)** | **MIC/μg.ml^-1^** | **Results** |
| --- | --- | --- | --- |
| Cefazolin |  | >=64.0 | R |
| Ceftazidime |  | >=64.0 | R |
| Ceftriaxone |  | >=64 | R |
| Aztreonam |  | >=64.0 | R |
| Gentamicin |  | >=16.0 | R |
| Tobramycin |  | >=16.0 | R |
| Compound sulfamethoxazole |  | ＜=20 | S |
| Cefepime |  | >=64 | R |
| Amikacin |  | >=64.0 | R |
| Imipenem |  | >=16.0 | R |
| Levofloxacin |  | >=8 | R |
| Ampicillin-sulbactam |  | >=32 | R |
| Ciprofloxacin |  | >=4 | R |
| Piperacillin-tazobactam |  | >=128.0 | R |
| Cefperazone-Sulbactam | 6 |  | R |
| Meropenem | 7 |  | R |
| Minocycline | 20 |  | S |
| Tigecycline | 23 |  | S |

S: susceptible; R: resistant； I: intermediate；MIC: minimum inhibitory concentration.

**Table S3.** Antimicrobial susceptibility of [*Escherichia*](javascript:;) [*coli*](javascript:;)

| **Antibiotics** | **K-B (mm)** | **MIC/μg.ml^-1^** | **Results** |
| --- | --- | --- | --- |
| Cefazolin |  | >=64.0 | R |
| Ceftazidime |  | >=64.0 | R |
| Ceftriaxone |  | >=64 | R |
| Aztreonam |  | >=64.0 | R |
| Gentamicin |  | >=16.0 | R |
| Compound sulfamethoxazole |  | ＜=20 | R |
| Cefepime |  | >=64 | R |
| Amikacin |  | >=64.0 | R |
| Imipenem |  | >=16.0 | R |
| Levofloxacin |  | >=8 | R |
| Ampicillin-sulbactam |  | >=32 | R |
| Ciprofloxacin |  | >=4 | R |
| Piperacillin-tazobactam |  | >=128.0 | R |
| Cefperazone-Sulbactam | 6 |  | R |
| Meropenem | 7 |  | R |
| Tobramycin | 8 |  | I |
| Minocycline | 20 |  | S |
| Tigecycline | 23 |  | S |

S: susceptible; R: resistant； I: intermediate；MIC: minimum inhibitory concentration.
